# Isolation and characterization of two porcine circovirus type 2 strains from Heilongjiang Province and Inner Mongolia Autonomous Region

**DOI:** 10.4142/jvs.25350

**Published:** 2026-05-27

**Authors:** Jintao Wang, Chun Lu, Guangqian Liu, Dongsong Guan, Yuan Shi, Mengyue Zhang, Zhishuan Ren

**Affiliations:** 1 College of Animal Science and Veterinary Medicine, Heilongjiang Bayi Agricultural University, Daqing 163319, China.; 2 Institute of Animal Science and Veterinary Medicine, Hei Longjiang Academy of Land Reclamation Sciences, Harbin 150038, China.; 3 Quality Testing Laboratory, Harbin 150008, China.

**Keywords:** PCV2d genotype, PK-15 cells, Rep protein, capsid epitopes, BALB/c mouse model

## Abstract

**Importance:**

Porcine circovirus type 2 (PCV2) impairs the immune function in pigs and contributes to immunosuppressive disease, causing substantial economic losses worldwide.

**Objective:**

To isolate and characterize PCV2 strains from two pig farms in China and evaluate pathogenicity in mice.

**Methods:**

Lymph node tissues suspected of a PCV infection were collected from farms in Heilongjiang Province and Inner Mongolia Autonomous Region. Virus isolation was performed in PK-15 cells and confirmed by a polymerase chain reaction, sequencing, indirect immunofluorescence, and transmission electron microscopy. The genotypes were assigned by phylogenetic analysis. The replication kinetics were evaluated by quantitative polymerase chain reaction, and the pathogenicity was assessed in BALB/c mice.

**Results:**

Two isolates were obtained and classified as PCV2d (PCV2-HLJ-92 and PCV2-NMG-103). The capsid amino acid sequences were identical between the isolates. Relative to the vaccine strains (PCV2a-LG and PCV2b-SH), both isolates carried multiple capsid amino acid substitutions. Regarding a PCV2d reference strain (BDH), a single substitution at residue 169 was observed. Differences in replication kinetics and tissue viral loads were observed between the isolates; these differences coincided with a Rep residue 6 substitution. Both isolates infected mice and produced histopathologic lesions primarily in the liver and lung.

**Conclusions and Relevance:**

These PCV2d isolates expand the available genomic and phenotypic data for circulating strains and may inform epidemiologic surveillance and vaccine development.

## INTRODUCTION

Porcine circovirus (PCV) is a single-stranded, non-enveloped DNA virus and a member of the genus Circovirus in the family Circoviridae. The PCV virion is one of the smallest DNA viruses capable of infecting mammals, with a diameter of approximately 17–20 nm [1,2,3,4,5,6]. Thus far, 5 genotypes of PCVs have been identified: PCV1, PCV2, PCV3, PCV4, and PCV5. PCV1 is generally considered non-pathogenic and was first identified as a contaminant in PK-15 cells [7]. PCV2 is often recognized as the main pathogen causing porcine circovirus-associated disease (PCVAD) [8]. PCV3 was first identified in the United States in 2016 [9]. It is frequently documented in coinfections with PCV2 and has been detected in multiple global regions [10,11]. PCV4 was first identified in China in 2019 [12], but no successful viral isolation has been confirmed to date. Liu et al. [5] recently discovered PCV5, a single-stranded circular DNA virus with a high prevalence in southern China, which is believed to be highly associated with PCVAD.

PCV2 was first isolated in 1998 from pigs exhibiting post-weaning multisystemic wasting syndrome [2,13], but a retrospective study reported that the PCV2 gene had been detected in samples collected in Germany in 1962 [14]. In addition, studies have shown that PCV2 may have originated a century ago [15]. In 2000, Lang et al. [16] first detected PCV2 in samples from 7 provinces and cities in China and speculated that it had already spread widely. Although PCV2 is a single-stranded DNA virus, it exhibits a remarkably high nucleotide substitution rate. Firth et al. [15] estimated this nucleotide substitution rate at 1.2 × 10^−3^ substitutions/site/year, indicating that the evolution speed of the virus is even close to that of single-stranded RNA viruses. Several studies have confirmed that PCV2 infects a range of hosts beyond swine, including ruminants, rodents, and canids [17,18,19]. These findings indicated potential for cross-species transmission of PCV2. An *in vitro* study showed that PCV2 could infect and replicate in human cell lines [20], underscoring its zoonotic potential. Previous research has indicated that PCV2 does not infect immunocompetent individuals, but it is unclear if it can infect immunocompromised individuals [21]. Therefore, the theoretical zoonotic risk of PCV2 cannot be disregarded.

The whole genome of PCV2 is approximately 1.7 kb and contains two major open reading frames (ORFs), ORF1 and ORF2. ORF1 is a conserved region situated on the positive strand of the PCV2 genome that serves as a common target for primer design in molecular diagnostics [22,23]. ORF2 exhibits high variability driven by immune selection pressure. Therefore, it is commonly used for phylogenetic analysis [24]. PCV2 can be divided into 8 genotypes based on the ORF2 sequence, namely PCV2a–PCV2h [25]. Wang et al. [11] reported a new genotype, PCV2i, in 2020. Currently, the PCV2a, PCV2b, and PCV2d strains are prevalent in China, with PCV2d strains dominant [26].

The replication-associated protein (Rep) and capsid protein (Cap) play critical roles in the PCV2 life cycle. The non-structural protein Rep is encoded by ORF1 and is essential for viral replication [27]. As the sole structural protein of PCV2 encoded by ORF2, Cap harbors immunodominant epitopes essential for antibody recognition [28]. Therefore, it is the primary target antigen for subunit vaccine design and serodiagnostic applications [29,30]. In addition, both ORF3 and ORF4 proteins induce apoptosis via distinct mechanisms. The ORF3 protein competes with p53 for Pirh2 binding, leading to p53 stabilization and apoptosis, whereas the ORF4 protein localizes to mitochondria via an interaction with ANT3 to trigger mitochondria-mediated apoptosis [31,32]. ORF5 protein promotes PCV2 replication in PK-15 cells by inducing autophagy [33], and the research on other proteins is relatively limited. This study isolated two PCV2d strains and conducted pathogenicity analysis, providing data to support and serve as a reference for understanding the characteristics of prevalent strains and vaccine development.

## METHODS

### Sample collection

In 2024, five lymph nodes suspected of a PCV infection were collected from nursery pigs on two farms. The pigs on both farms were immunized with the commercial PCV2 vaccine. Three samples from a 500-sow farm in the Inner Mongolia Autonomous Region, where the pigs exhibited fever, cough, anorexia, emaciation, and skin rashes. The other two samples were collected from a 1,000-sow farm in Heilongjiang Province, where the affected pigs showed cough, anorexia, skin rashes, and enlarged inguinal lymph nodes. The samples were stored at −80°C for use. The lymph nodes were collected from pigs euthanized by farm staff in accordance with farm standard operating procedures under the oversight of the study protocol. After the samples were collected, the animals were transferred to the harmless treatment center for treatment.

### Primer design and synthesis

The polymerase chain reaction (PCR) primers used to detect PCV2 were from GB/T 21674-2008 [34], and the quantitative polymerase chain reaction (qPCR) primers used to detect PCV2 were from GB/T 34745-2017 (Table 1) [35]. The nucleotide sequences of the PCV2 whole genome were downloaded from National Center for Biotechnology Information on 15 February 2026 (Supplementary Table 1) and compared using MegAlign 7.1.0 software (DNASTAR, USA). The primers of the PCV2 whole genome (Table 1) were designed by Oligo 7.60 software (Molecular Biology Insights, USA). All primers were synthesized by Sangon Biotech (Shanghai) (China).

**Table 1:** Primer sequences

Purpose	Primers	Sequences (5’-3’)	Product length/bp
PCR detection	P1-F	CCGCGGGCTGGCTGAACTT	1,154
	P1-R	ACCCCCGCCACCGCTACC	
qPCR detection	P2-F	GGGCCAGAATTCAACCTTAACC	171
	P2-R	CGCACCTTCGGATATACTGTCA	
Whole genome amplification	P3-F	AGCACCTCAGCAGCAACAT	1,253
	P3-R	TAACCCAGCCCTTCTCCTACC	
	P4-F	GCCAGTTCGTCACCCTTTCC	1,256
	P4-R	GGTACTCACAGCAGTAGAGAGG	

PCR, polymerase chain reaction; qPCR, quantitative polymerase chain reaction; PCV2, porcine circovirus type 2.

### Sample preparation and PCR amplification

A 200 mg tissue sample was ground in liquid nitrogen. The resulting tissue homogenate was prepared with 800 µL phosphate-buffered saline (PBS) in a 2.0 mL sterile EP tube and stored at −80°C. DNA was extracted using a TIANamp Genomic DNA Kit (TIANGEN, China). The PCR reaction system of PCV2 was 50 µL in volume, including 25 μL Premix Taq (Takara Bio, China), 2.5 µL of forward and reverse primers each, 5 μL of template, and 15 μL of ddH_2_O. The reaction conditions were as follows: pre-denaturation at 94°C for 1 min; denaturation at 98°C for 10 sec; 35 cycles of annealing at 62°C for 45 sec, extension at 72°C for 45 sec; extension at 72°C for 10 min. PCR products were detected by 1.0% agarose gel electrophoresis. The results were analyzed using a full-automatic gel imaging analysis system (Bio-Rad, USA).

### Isolation and culture of PCV2

Chloroform (50 μL) was combined with 950 μL of PCV2-positive tissue homogenate for 10 min, and centrifuged at 3,000 rpm/min at 4°C for 10 min. The supernatant was transferred to a new sterile 2 mL EP tube and filtered through a 0.22 μm filter (Biosharp, China). Subsequently, 1% penicillin–streptomycin (Solarbio, China) was added, and the sample was stored at −80°C. PCV1-free PK-15 cells stored in the laboratory were cultured in T-25 flasks for a subsequent PCV2 infection. PK-15 cells with a density of 70% were washed twice with PBS, inoculated with 2 mL of the processed tissue homogenate, and incubated at 37°C in an atmosphere containing 5% CO_2_ for 2 h. The cell supernatant was discarded and replaced with a maintenance medium, consisting of Dulbecco’s Modified Eagle Medium (DMEM, Gibco, USA), 2% fetal bovine serum (Clark Bioscience, USA), and 1% penicillin–streptomycin (Solarbio). The cells were then incubated at 37°C in an atmosphere containing 5% CO_2_. After 48 h, the cells were frozen and thawed three times at −80°C. The first passage virus was collected and re-inoculated into PK-15 cells. After five consecutive passages, PCR was performed to identify the virus.

### Growth curve drawing of PCV2

The two isolated strains were respectively inoculated into PK-15 cells in six-well plates. The cells were collected at 24, 48, 72, and 96 h after inoculation, and DNA was extracted. qPCR with the 2 × Universal SYBR Green Fast qPCR Mix (ABclonal, China) was performed on the Gentier 96R Real-Time PCR System (Tianlong, China) to detect the viral load at each time point. A standard curve was generated using the recombinant plasmid pUC18-PCV2-L2.

### Indirect immunofluorescence identification of PCV2

PK-15 cells grown on 12-well plates were infected with PCV2 for 48 h, fixed with 4% paraformaldehyde at room temperature for 30 min, and treated with 0.2% Triton X-100 at room temperature for 10 min. Subsequently, the cells were blocked with PBS containing 5% bovine serum albumin (Seven-Bio, China) for 30 min. The primary antibody was a commercially available mouse anti-PCV2 Cap-specific monoclonal antibody (PhyCell Medical Research, China). The sample was incubated at 4°C for 12 h, followed by Alexa Fluor 594-conjugated goat anti-mouse immunoglobulin G (ZSGB-BIO, China) at room temperature for 1.5 h. The cells were counterstained with 4,6-diamidino-2-phenylindole (Biosharp) at room temperature for 5 min. The fluorescence was visualized using an inverted fluorescence microscope (IX 73, Olympus, Japan). The titers of the two isolates were both 1 × 10^4.75^ TCID50/mL by immunofluorescence assay (IFA) and the Reed-Muench method.

### Transmission electron microscopy (TEM) observation of PCV2

The virus liquid was centrifuged at 12,000 rpm/min at 4°C for 30 min, and the supernatant was collected in sterile 2.0 mL EP tubes. A 1 μL sample of PCV2 monoclonal antibody (PhyCell Medical Research) was added to 300 μL of viral supernatant, and the mixture was incubated at 37 °C for 30 min. The mixed liquid was negatively stained with 2% phosphotungstic acid (Solarbio) and examined using TEM (JEOL, Japan).

### Genome amplification and sequence analysis of PCV2

The whole-genome sequences of the two isolates were amplified by PCR using the specific primers (Table 1). The PCR products were sequenced by Sangon Biotech (Shanghai). The complete genome sequences of the two isolates were submitted to GenBank. Homology analysis and phylogenetic tree construction for the ORF2 gene and whole genome of the two isolates were performed using MegAlign 7.1.0 software (DNASTAR) and MEGA 11.0.13 software (https://www.megasoftware.net). The resulting data were then visualized using GraphPad Prism 9.5.0 software (Graphpad Software, USA) and ITOL (https://itol.embl.de).

### Amino acid sequence analysis of PCV2

The ORF1 and ORF2 nucleotide sequences of the two isolates were translated into the amino acid sequences of the Rep and Cap, respectively, using MEGA 11.0.13 software (https://www.megasoftware.net). The resulting amino acid sequences were then visualized using the Jalview 2.11.5.1 software (https://www.jalview.org).

### Pathogenicity studies in mice

The pathogenicity of the isolated strains was evaluated by randomly allocating 54 female BALB/c mice (6-week-old) with comparable body weights into three groups: the control group, the PCV2-HLJ-92 challenge group, and the PCV2-NMG-103 challenge group. The mice were housed under specific pathogen-free conditions (22°C ± 2°C, 50%–60% humidity, 12 h light/dark cycle) with free access to sterilized food and water. The mice in the challenge groups were injected intraperitoneally with 200 μL of virus suspension containing 1 × 10^4.75^ TCID50/mL, and the control mice received an equivalent volume of DMEM medium. The experimental period was 35 days, with 5 time points (7, 14, 21, 28, and 35 days). Six mice from each group were selected randomly for body weight measurements at every time point. For viral load and pathological analysis, three mice from each group were randomly selected and euthanized via cervical dislocation for the collection of the heart, liver, spleen, lung, kidney, and brain at every time point. Each tissue sample was divided into two portions; one was fixed in 4% paraformaldehyde for histopathology, and the other was stored at −80°C for subsequent viral load quantification.

Frozen tissue samples were homogenized in sterile PBS. The total DNA was extracted using the TIANamp Genomic DNA Kit (TIANGEN). The viral loads of the isolates in the tissues were measured by qPCR. Tissues exhibiting higher viral loads were embedded in paraffin, and 4 μm-thick sections were prepared. The sections were stained with hematoxylin and eosin for the histopathological analysis.

### Statistical analysis

The GraphPad Prism 9.5.0 software (GraphPad Software) was used for statistical analysis. The data are presented as the mean ± SD, and the results were compared by two-way analysis of variance with a Sidak correction. In all analyses, *p* values < 0.05 were considered statistically significant.

## RESULTS

### Isolation and identification of PCV2

**Fig. 1:**
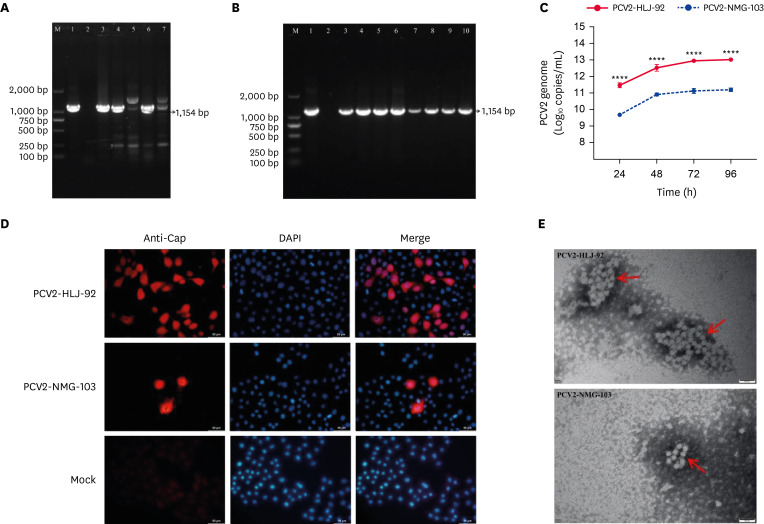
Isolation and identification of PCV2. (A) PCV2 detection in the clinical samples (M: DL2000 DNA Marker; 1: positive control; 2: negative control; 3–7: clinical samples). (B) PCV2 detection in different generation cells (M: DL2000 DNA Marker; 1: positive control; 2: negative control; 3–6: PCV2-HLJ-92 at the 5^th^, 10^th^, 15^th^, and 20^th^ passages; 7–10: PCV2-NMG-103 at the 5^th^, 10^th^, 15^th^, and 20^th^ passages). (C) Growth curve of two PCV2 isolates. Data are presented as the mean ± SD (n = 3 per group per time point). (D) Indirect immunofluorescence assays of the two isolates. PCV2 Cap was red (Alexa Fluor 594), and DAPI was blue. Scale bar =  50 μm. Magnification, ×40. (E) Electron microscopy images of the two isolates. Scale bar =  50 nm. Magnification, ×80,000. PCV2, porcine circovirus type 2; DAPI, 4′,6-diamidino-2-phenylindole. ^****^p < 0.0001.

PCV2 was positive in the clinical samples (Fig. 1A), and the virus could be detected continuously across different passages (Fig. 1B). The two isolates showed a stable replication period from 24 to 72 h. They entered the plateau period after 72 h (Fig. 1C). IFA showed that the isolated virus could specifically bind to the anti-PCV2 Cap monoclonal antibody (Fig. 1D). TEM showed that the enriched virus exhibited spherical particles with a diameter of approximately 17 nm (Fig. 1E). Based on the above results, two PCV2 strains were isolated and called PCV2-HLJ-92 and PCV2-NMG-103.

### Nucleotide and amino acid sequence analysis of PCV2

**Fig. 2:**
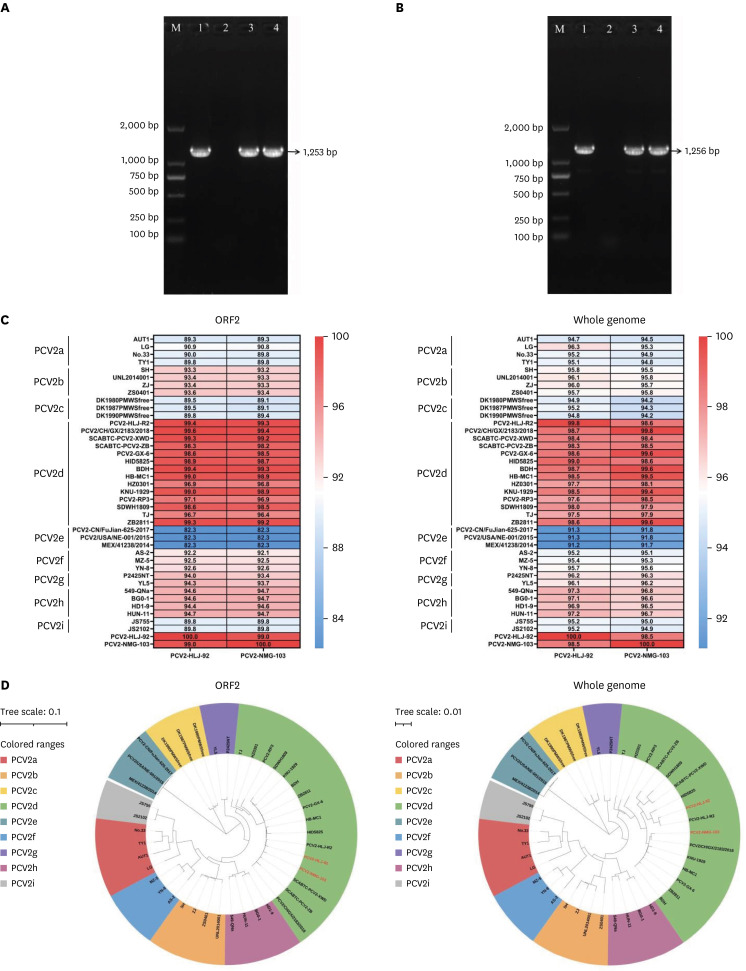
ORF2 and whole genome sequence analysis of PCV2. (A) Results of segmented amplification using L1F/R (M: DL2000 DNA Marker; 1: positive control; 2: negative control; 3: PCV2-HLJ-92; 4: PCV2-NMG-103). (B) Results of segmented amplification using L2F/R (M: DL2000 DNA Marker; 1: positive control; 2: negative control; 3: PCV2-HLJ-92; 4: PCV2-NMG-103). (C) Homology analysis of PCV2 ORF2 and whole genome. (D) Phylogenetic tree analysis based on PCV2 ORF2 and the whole genome. The Neighbor-Joining tree was constructed with 1,000 bootstrap replicates. ORF, open reading frame; PCV2, porcine circovirus type 2.

The whole genomes of the PCV2-HLJ-92 and PCV2-NMG-103 were amplified by PCR into two segments (Fig. 2A and B). These amplified fragments were sequenced and spliced to obtain the whole genome sequences of both isolates. Homology (Fig. 2C) and phylogenetic analyses (Fig. 2D) showed that both isolates belonged to the PCV2d genotype. The two isolates shared 99% ORF2 sequence homology and 98.5% whole genome homology. Their ORF2 sequence homology to the PCV2d reference strains ranged from 96.4% to 99.6%, and the entire genome sequence homology to the PCV2d reference strains ranged from 97.5% to 99.8%.

**Fig. 3:**
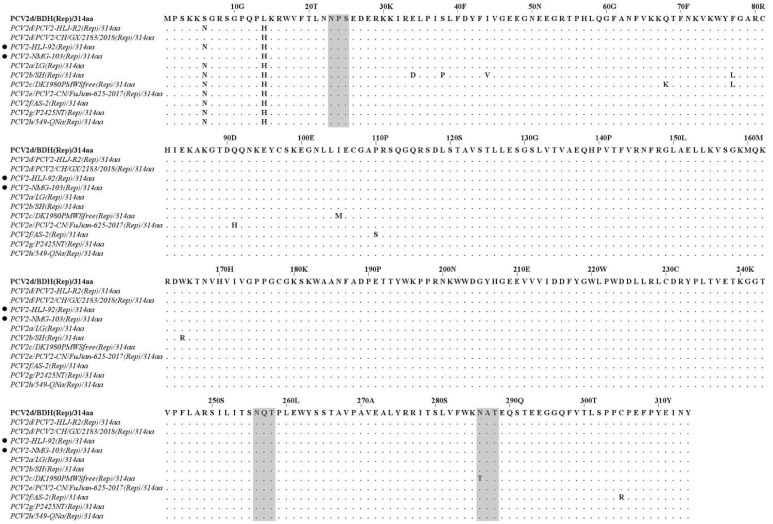
Amino acid sequence analysis of PCV2 Rep. The grey highlighted area: N-glycosylation site; the PCV2 isolates identified in this study are labeled with black circles. PCV2, porcine circovirus type 2.

The amino acid sequence analysis of the Rep (Fig. 3) revealed high sequence conservation. The two isolates differed by only one amino acid at position 6 in the Rep protein. Compared to the vaccine strain LG (PCV2a), PCV2-HLJ-92 had an identical Rep sequence, whereas PCV2-NMG-103 had a single amino acid substitution at residue 6. In contrast, both isolates carried multiple amino acid mutations relative to the vaccine strain SH (PCV2b) at Rep residues 34, 38, 44, 77, and 165. Compared to the PCV2d reference strain BDH, both isolates carried a mutation at residue 14, and only PCV2-HLJ-92 had a mutation at residue 6.

**Fig. 4:**
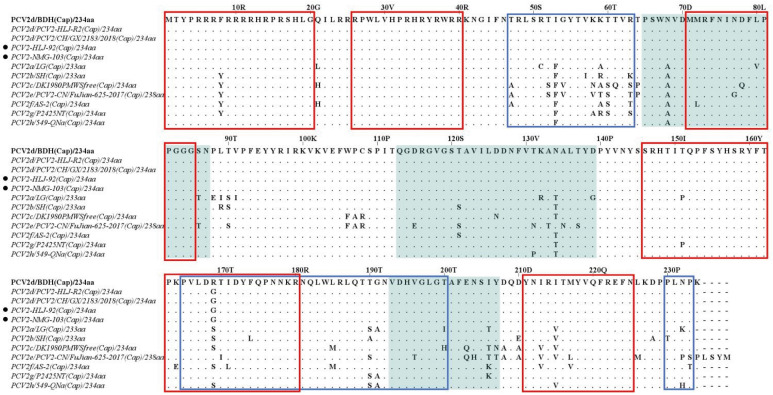
Amino acid sequence analysis of PCV2 Cap. Green highlighted area: linear B-cell epitopes; blue box: conformational epitopes; red box: T-cell epitopes; the PCV2 isolates identified in this study are labeled with black circles. PCV2, porcine circovirus type 2.

The Cap amino acid sequences of both PCV2 isolates were identical to those of the PCV2d reference strains PCV2-HLJ-R2 and PCV2/CH/GX/2183/2018 (Fig. 4, Supplementary Table 2). Compared to the PCV2d strain BDH, only one mutation was detected at residue 169. Multiple amino acid variations were observed within linear B-cell epitopes and conformational epitopes of the Cap in both isolates relative to other reference strains. Specifically, compared to the vaccine strain LG (PCV2a), mutations occurred at residues 51, 53, 59, 68, 80, 86, 132, 134, 139, 169, 190, 191, 200, 206, and 232. Compared to the vaccine strain SH (PCV2b), mutations were located mainly at residues 53, 57, 59, 63, 68, 121, 134, 169, 174, 190, and 230. Furthermore, both isolates had fewer mutations in the T-cell epitopes. They differed from the LG (PCV2a) vaccine strain at residues 80, 151, 169, and 215, and the SH (PCV2b) vaccine strain at residues 8, 169, 174, and 215.

### Pathogenicity analysis of PCV2 in mice

**Fig. 5:**
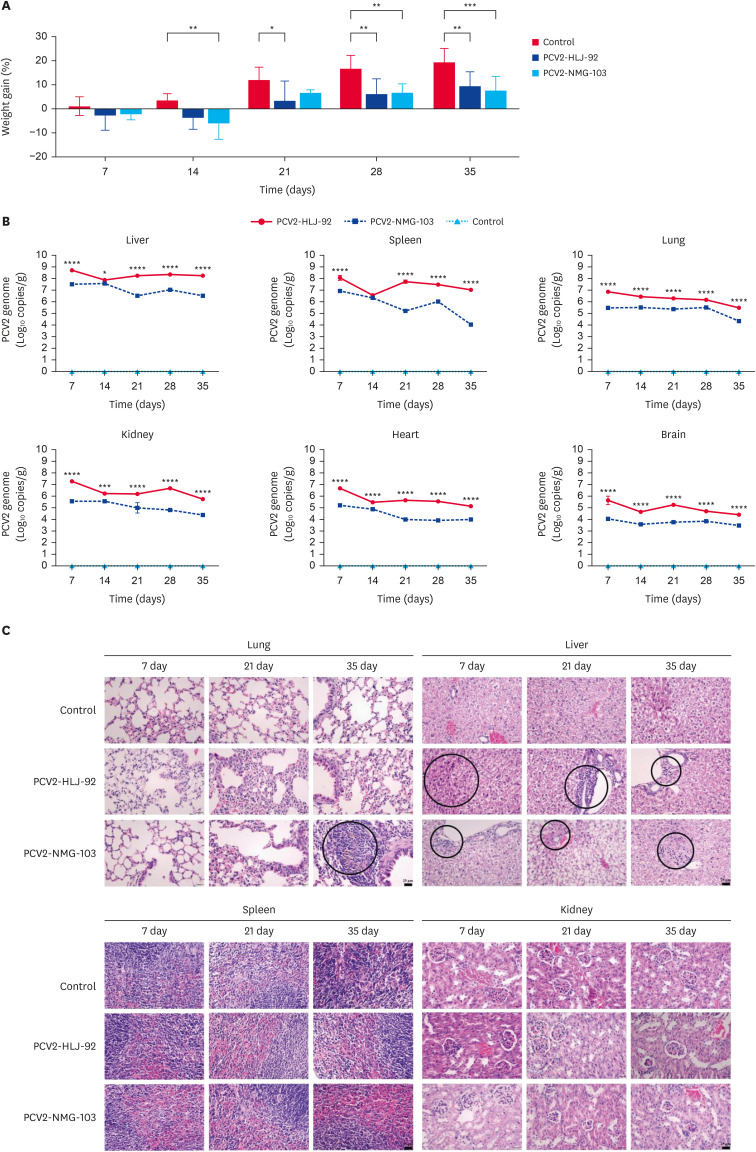
Pathogenicity analysis of PCV2 in mice. (A) Body weight changes of mice (n = 6 per group per time point). Data are presented as mean ± SD. (B) Tissue viral load in mice from different groups (n = 3 per group per time point). The data are presented as the mean ± SD. Statistical comparisons were performed only between the two challenged groups (PCV2-HLJ-92 vs PCV2-NMG-103). (C) Pathological observation of different tissues in mice. The pathological tissue sections were stained with hematoxylin and eosin. Scale bar = 25 μm. Magnification, ×400. PCV2, porcine circovirus type 2. ^*^p < 0.05, ^**^p < 0.01, ^***^p < 0.001, ^****^p < 0.0001.

No obvious clinical signs or mortality were observed in the challenged groups throughout the study. Body weight monitoring (Fig. 5A) suggested that the mice in both challenged groups exhibited a decreasing trend compared to the control group during the first 14 days. From days 14 to 35, the body weight of the challenged mice gradually recovered but remained lower than that of the control group. Fig. 5B shows the viral loads in different tissues. The highest viral loads were observed in the liver, while lower levels were detected in the heart and brain. The viral loads in the tissues of the two challenged groups declined gradually over time, and those of the PCV2-HLJ-92 group were significantly higher than those in the PCV2-NMG-103 group.

A histopathological examination (Fig. 5C) revealed the lesions primarily in the lung and liver. In the lungs, the mice in the two challenged groups did not exhibit significant pathological damage on day 7, but alveolar wall thickening was observed in all infected mice on days 21 and 35. Furthermore, the bronchus-associated lymphoid tissue (BALT) hyperplasia and macrophages containing brownish-yellow granules within the hyperplastic BALT were observed only in the PCV2-NMG-103 group on day 35. In the liver, the mice in the PCV2-HLJ-92 group exhibited multifocal necrosis on day 7, and inflammatory cell infiltration in the portal areas on days 21 and 35. The mice in the PCV2-NMG-103 group showed mild lymphocytic infiltration in the portal areas on day 7, focal necrosis in the portal area on day 21, and multiple lymphoid foci on day 35. The spleens of the mice in both challenge groups showed dilation and congestion of the red pulp sinuses, whereas no significant lesions were observed in the kidneys.

## DISCUSSION

PCV2 is a globally prevalent virus that induces immunosuppression in pigs, predisposes them to secondary infections, and causes substantial economic losses to the swine industry annually [36,37]. Consistent with its widespread circulation, continuous surveillance of PCV2 field strains is essential for monitoring viral evolution and informing disease control strategies. In this context, two PCV2 strains, designated PCV2-HLJ-92 and PCV2-NMG-103, were isolated from Heilongjiang Province and Inner Mongolia Autonomous Region. Homology and phylogenetic analyses confirmed that both isolates belonged to the PCV2d genotype, which was consistent with the currently epidemic trend of PCV2 in China. Phylogenetic analysis also showed that the two isolates were closely related to PCV2d reference strains uploaded from China in recent years, indicating that they may share a common ancestor. The two isolates were passaged stably over 20 times in PK-15 cells, showing that they could proliferate stably *in vitro*.

The PCV2 Rep is an important non-structural protein that is critical for viral replication [27]. The results of this study showed that compared to the reference strain BDH (PCV2d), the PCV2-HLJ-92 strain harbored an S6N mutation in the Rep. Specifically, Wu et al. [38] reported that the S6N mutation significantly enhanced the replication efficiency of PCV2d strains in cultured cells. This may have accounted for the higher viral titer of PCV2-HLJ-92 compared to PCV2-NMG-103 observed in the virus growth curve. Cap is the sole structural protein of PCV2, which contains multiple antigenic epitopes and serves as the primary target for host immune responses [28,39]. Studies have shown that PCV2a and PCV2b-based vaccines conferred cross-protection against a PCV2d challenge [40,41], but the evidence indicates that homologous vaccines provided significantly higher protection against matched genotypes than heterologous genotypes [42]. Compared to the vaccine strains LG and SH, two isolates exhibited multiple amino acid substitutions within the linear and conformational B-cell epitopes. These mutations might compromise the antibody recognition efficiency, thereby diminishing the protective efficacy of current vaccines against field-prevalent isolates.

Nevertheless, these findings require further experimental validation. Furthermore, compared to the reference strain BDH isolated from Heilongjiang Province in 2008, the two isolates exhibited only a single mutation at the 169^th^ amino acid residue (R169G). Studies by Lv et al. [43] and Leng et al. [44] have also documented the variation at this residue in PCV2d strains. These findings suggest that position 169 of the Cap may have become a high-frequency mutation hotspot in PCV2d, and this mutation was likely driven by immune selection pressure. Cellular immunity plays a critical role in viral clearance [45,46]. Compared with the B-cell epitopes, the isolates exhibited fewer mutations in the T-cell epitopes, highlighting their potential as targets for broad-spectrum vaccines. Collectively, the impact of amino acid mutations in the Rep and Cap of the isolates on viral pathogenicity and immunogenicity remains to be developed.

Mouse models are a widely used tool for studying viral replication and pathogenesis *in vivo* [47]. Consequently, this study used BALB/c mice as the experimental model to investigate PCV2 pathogenicity. In this study, infection with either PCV2 isolate induced transient fluctuations in the body weights of the mice, characterized by an initial decrease followed by recovery. This pattern suggests that the growth of the host is affected by a viral infection, which is consistent with the report by Jiao et al. [48]. Concurrently, qPCR analysis showed that both strains replicated efficiently in all examined tissues. The viral loads in these tissues generally declined gradually over time. These findings suggest that the isolated PCV2d strains can establish a productive infection in mice, which was then controlled and progressively cleared by the host’s immune response. In the present study, PCV2-HLJ-92 showed higher replication kinetics *in vitro* and viral loads *in vivo* than PCV2-NMG-103. This result suggests that the difference at Rep residue 6 was associated with the viral replication levels, consistent with a prior report [38]. Histopathological analysis revealed time-dependent and tissue-specific lesions in the mice infected with two PCV2d isolates. In both challenged groups, inflammatory cell infiltration in hepatic portal areas and the high viral loads in this tissue confirmed the high tropism of both isolates for liver tissue, which is basically consistent with Guo et al. [49]. In the lungs, peribronchial and alveolar wall thickening—a characteristic histopathological feature of interstitial pneumonia—was observed in both challenged groups during the later stages of infection, indicating inflammatory pathological changes induced by PCV2. Furthermore, dilation and congestion of the red pulp sinusoids were observed in the spleens of mice from both infected groups, which might reflect immune activation and hemodynamic alterations in this key peripheral immune organ in response to viral infection. Although high viral loads were detected in the kidney, no significant pathological lesions were observed. This suggested that the virus presence in the kidney may be insufficient to cause significant histological damage.

Overall, this study isolated two PCV2d strains, PCV2-HLJ-92 and PCV2-NMG-103. The findings suggested that the difference at Rep residue 6 was associated with higher viral replication *in vitro* and *in vivo*. The frequent amino acid mutations observed in the critical epitopes of Cap suggest that these changes may help reduce the protection of current vaccines. Pathogenicity studies confirmed that both isolates replicated efficiently in BALB/c mice and induced significant pathological lesions primarily in the lung and liver. In contrast, only mild lesions were observed in the spleen, with no notable damage observed in the kidney. These results enhance the understanding of PCV2d evolution and pathogenesis and provide valuable genetic evidence for the development of next-generation vaccines.

## SUPPLEMENTARY MATERIALS

Supplementary Table 1 The GenBank accession numbers for the PCV2 reference strain

Supplementary Table 2 Main amino acid mutation site of PCV2 Cap
